# Editorial

**Published:** 1915-01

**Authors:** 


### EDITORIAL

The Business Office of the *Journal-Record* is 23 West Alabama Street.
**The Editorial Office is 1014-15 Atlanta National Bank Building.**
Address all Business Communications to *Journal-Record of Medicine*, 23 West Alabama Street.
**Make remittances payable to *The Journal-Record of Medicine*.**
**On matters pertaining to the Editorial and Original Communications, address Edgar G. Ballenger, M. D., Atlanta, Ga.**
**Reprints of Original Articles will be furnished at cost price. Requests for the same should always be made in THE MANUSCRIPT.**
**We will present, postpaid, on request to each contributor of an original article, twenty (20) marked copies of *The Journal-Record of Medicine* containing such article.**

### SOME ASPECTS OF LONG BATTLES.

There are not many things reported to the medical or surgical world as yet from the seat of war that were not to be expected, but two have attracted attention.

The first is that many soldiers have broken down with severe nervous troubles and others have become insane when they have neither been wounded nor starved. At first it was thought that a new sort of timidity, not to say cowardice, was showing itself and that the sufferers were attempting to evade duty. But this soon proved untrue. The real reason was that the men could not withstand the terrible noises of their own cannon and the bursting of the enemies' shells near them. Never before in the history of the world has there been anything like this kind of strain put upon men. They have had to withstand not a few hours or at most a day of it as they might have had in other wars, but in many places they have it day and night for months until the wear and tear have become unbearable. The results were apparently unforeseen by the officers and men had to remain there under the torture by endless noise until the authorities learned through their going insane that they would have to be relieved. We read that the Germans are regularly relaying their men at the trenches and that one shift has a chance to rest away from the front while the other works.

The men insane from the noise appear to recover quickly when rested at the rear, and they are soon eager to return to the lines showing that cowardice has no part in the matter.

The second peculiar effect to be noted is that of shells bursting near men and killing them without any part of the missile striking them. Shock was said to be the cause of death but that did not mean anything. It evidently was not fright because the shells were bursting close enough at many times to make them familiar but not to harm.

A recent observation disclosed the reason. A soldier was very near to a large shell when it burst and though he was not struck, he found himself incapacitated and he could not breathe well.

He attempted to return to his work but soon died. Autopsy revealed that the sudden great pressure of the air caused by the bursting of the shell had so compressed the air within his lungs as to rupture the walls of the bronchioles and to fill the lungs with blood. R. R. D.

